# Non-Gaussian diffusion metrics with whole-tumor histogram analysis for bladder cancer diagnosis: muscle invasion and histological grade

**DOI:** 10.1186/s13244-024-01701-z

**Published:** 2024-06-09

**Authors:** Zhichang Fan, Junting Guo, Xiaoyue Zhang, Zeke Chen, Bin Wang, Yueluan Jiang, Yan Li, Yongfang Wang, Guoqiang Yang, Xiaochun Wang

**Affiliations:** 1 Department of Radiology, The First Hospital of Shanxi Medical University, Taiyuan, Shanxi China; 2 Department of Medical Imaging, Shanxi Medical University, Taiyuan, Shanxi China; 3 Department of MR Research Collaboration, Siemens Healthineers, Beijing, China

**Keywords:** Diffusion-weighted imaging, Bladder cancer, Muscle invasion, Histological grade

## Abstract

**Purpose:**

To investigate the performance of histogram features of non-Gaussian diffusion metrics for diagnosing muscle invasion and histological grade in bladder cancer (BCa).

**Methods:**

Patients were prospectively allocated to MR scanner1 (training cohort) or MR2 (testing cohort) for conventional diffusion-weighted imaging (DWI_conv_) and multi-*b*-value DWI. Metrics of continuous time random walk (CTRW), diffusion kurtosis imaging (DKI), fractional-order calculus (FROC), intravoxel incoherent motion (IVIM), and stretched exponential model (SEM) were simultaneously calculated using multi-*b*-value DWI. Whole-tumor histogram features were extracted from DWI_conv_ and non-Gaussian diffusion metrics for logistic regression analysis to develop diffusion models diagnosing muscle invasion and histological grade. The models’ performances were quantified by area under the receiver operating characteristic curve (AUC).

**Results:**

MR1 included 267 pathologically-confirmed BCa patients (median age, 67 years [IQR, 46–82], 222 men) and MR2 included 83 (median age, 65 years [IQR, 31–82], 73 men). For discriminating muscle invasion, CTRW achieved the highest testing AUC of 0.915, higher than DWI_conv_’s 0.805 (*p* = 0.014), and similar to the combined diffusion model’s AUC of 0.885 (*p* = 0.076). For differentiating histological grade of non-muscle-invasion bladder cancer, IVIM outperformed a testing AUC of 0.897, higher than DWI_conv_’s 0.694 (*p* = 0.020), and similar to the combined diffusion model’s AUC of 0.917 (*p* = 0.650). In both tasks, DKI, FROC, and SEM failed to show diagnostic superiority over DWI_conv_ (*p* > 0.05).

**Conclusion:**

CTRW and IVIM are two potential non-Gaussian diffusion models to improve the MRI application in assessing muscle invasion and histological grade of BCa, respectively.

**Critical relevance statement:**

Our study validates non-Gaussian diffusion imaging as a reliable, non-invasive technique for early assessment of muscle invasion and histological grade in BCa, enhancing accuracy in diagnosis and improving MRI application in BCa diagnostic procedures.

**Key Points:**

- Muscular invasion largely determines bladder salvageability in bladder cancer patients.
- Evaluated non-Gaussian diffusion metrics surpassed DWI_conv_ in BCa muscle invasion and histological grade diagnosis.
- Non-Gaussian diffusion imaging improved MRI application in preoperative diagnosis of BCa.

**Graphical Abstract:**

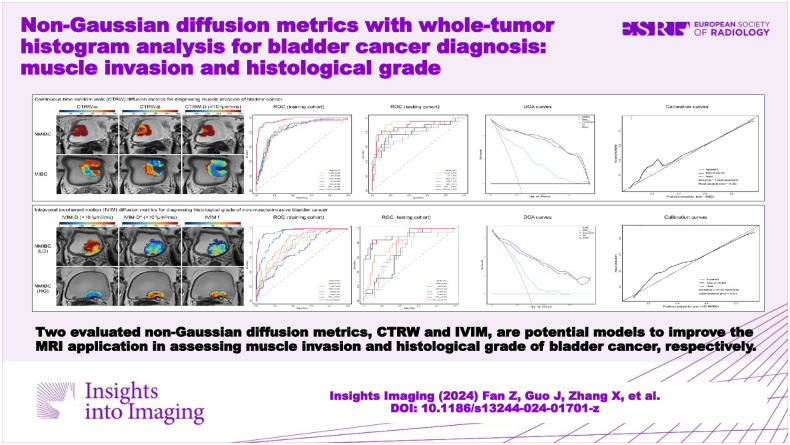

## Introduction

Bladder cancer (BCa) is a significant global health concern, ranking as the 10th most commonly diagnosed cancer worldwide and carrying the highest per-patient lifetime treatment costs [1, 2]. Precision clinical management of BCa depends highly on evaluating muscle invasion and histological grade, categorizing it into three major risk groups: low-grade non-muscle-invasive bladder cancer (LG-NMIBC), high-grade (HG)-NMIBC, and muscle-invasive bladder cancer (MIBC), each requiring distinct treatment strategies [3–5]. Muscle invasion is the foremost consideration for urologists when determining the feasibility of bladder preservation in patients [3, 4]. Subsequently, histological grade guides assessments for repeat resection, follow-up frequency, and treatment intensity in NMIBC patients [3, 5, 6]. Hence, accurate preoperative staging and grading are crucial for precision medicine and optimal resource allocation [7]. Transurethral resection of bladder tumor (TURBT) and MRI are standard procedures for preoperative assessment. However, TURBT is invasive with risks of understaging and undergrading [8, 9]; conversely, MRI often encounters overstaging and can’t directly assess histological grade [10, 11].

Diffusion-weighted imaging (DWI) emerges as a promising tool for quantifying tumor microstructure [12–14]. Conventional DWI (DWI_conv_) is a Gaussian diffusion model, assuming that the probability of water molecules moving in any direction within tissues is equal [12]. Thus, the Gaussian assessment of DWI is applicable to microenvironments with simple structures. Conversely, non-Gaussian diffusion models posit unequal movement of water molecules across directions due to obstacles like cell membranes, fibers, and vascular walls [12, 14]. Thus, the non-Gaussian assessment of DWI is more applicable to actual microenvironments with complex structures. To better connect diffusion model metrics with biologically pertinent microstructures, many non-Gaussian diffusion models, like intravoxel incoherent motion (IVIM), diffusion kurtosis imaging (DKI), stretched exponential model (SEM), fractional-order calculus (FROC), and continuous time random walk (CTRW), have been developed. In a recent study [15], the FROC has outperformed DWI_conv_ in diagnosing muscle invasion (accuracy, 78% vs. 69%) and histological grade (accuracy, 88% vs. 74%).

However, the theoretical advantages of non-Gaussian diffusion models have never been extensively validated in BCa clinical practice. Thus, we aim to compare the diagnostic performances of non-Gaussian diffusion models and DWI_conv_ for muscle invasion and histological grade in BCa.

## Materials and methods

### Study participants

**Fig. 1:**
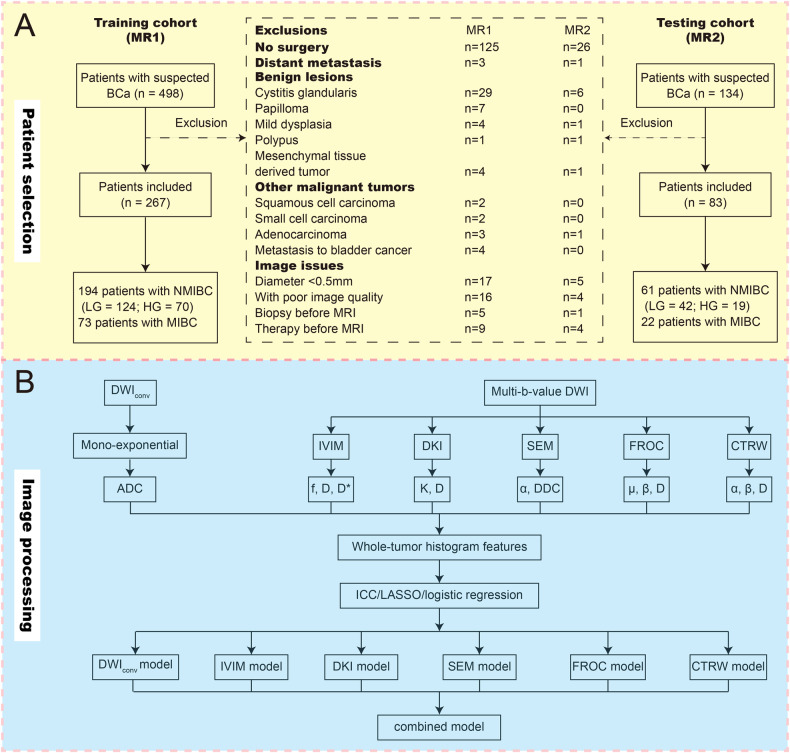
**A** Flowchart of participants selection and (**B**) pipeline of image post-processing and histogram analysis. NMIBC, non-muscle-invasive bladder cancer; MIBC, muscle-invasive bladder cancer; LG, low-grade; HG, high-grade; DWI_conv_, conventional diffusion-weighted imaging; ADC, apparent diffusion coefficient; IVIM, intravoxel incoherent motion; DKI, diffusion kurtosis imaging; SEM, stretched exponential model; FROC, fractional-order calculus; CTRW, continuous time random walk; ICC, intraclass correlation coefficient; LASSO, least absolute shrinkage and selection operator

This prospective study (No. K-K112) received approval from our hospital’s ethics committee and consecutively involved a total of 632 participants with suspected bladder tumors from January 2022 to July 2023. Informed consent was obtained prior to MRI examinations. Participants were randomly assigned to two MRI scanners (MR1: training cohort; MR2: testing cohort) for multi-*b*-value scanning with a ratio of 4:1 [16]. Inclusion criteria mandated pathological confirmation (TURBT or radical cystectomy) of non-metastatic urothelial carcinoma within two weeks following the MRI examination. Exclusion criteria and process of participant selection are shown in Fig. 1A. According to pathological results [17, 18], all included patients were divided into three groups: MIBC (pT stage ≥ 2), HG-NMIBC and LG-NMIBC.

### MRI parameters

To ensure sufficient bladder distension, participants were instructed to empty their bladder 1–2 h before the MRI examination, avoiding drinking or urinating until the scan was completed. Multi-*b*-value DWI (11 *b* values of 0, 50, 100, 150, 200, 500, 800, 1000, 1500, 2000, and 2500 sec/mm^2^) was performed after routine scan sequences of T1-weighted, T2-weighted, conventional diffusion-weighted (b_0_: 0 sec/mm^2^; b_1_: 800 sec/mm^2^) and dynamic contrast-enhanced (DCE). Multi-*b*-value DWI acquisition was conducted using an integrated slice-specific dynamic shimming single-shot echo planar imaging (SS-EPI) sequence on a 3 T scanner MR1 (MAGNETOM Vida, Siemens Healthcare), with the acquisition time of 11 min 30 s, or using a multi-slice SS-EPI sequence on one another 3 T scanner MR2 (MAGNETOM Skyra, Siemens, Healthcare), with the acquisition time of 8 min. All parameters of imaging acquisition were documented in Appendix Table S1, S2.

### Image processing

The metric map of DWI_conv_, apparent diffusion coefficient (ADC), was generated on the scanner console with a mono-exponential formula [12]: $S={S}_{0}\exp \left(-{bADC}\right)$, where S represents the diffusion-weighted signal and S_0_ represents the signal intensity without diffusion weighting, ADC reflects the average diffusion rate.

A total of 13 metrics of five non-Gaussian diffusion models, including (1) f, D and D* metrics of IVIM model; (2) K and D metrics of DKI model; (3) α and DDC metrics of SEM model; (4) β, μ and D metrics of FROC model; (5) α, β and D metrics of CTRW model, were simultaneously generated from multi-*b*-value data using an in-house developed software (BoDiLab) based on the open-source Python toolkit DIPY (https://dipy.org). Details were as follows [12, 14, 15]:

1. $S={S}_{0}\left[f\exp \left(-b{D}^{* }\right)+\left(1-f\right)\exp \left(-{bD}\right)\right]$ for the IVIM, where f is the volume fraction of the vasculature, D* reflects the diffusion in microvasculature, and D reflects the pure diffusion rate without perfusion;
2. $S={S}_{0}\exp \left[-{bD}+\frac{1}{6}K{\left({bD}\right)}^{2}\right]$ for the DKI, where K reflects the complexity of tissue microstructures, D reflects the diffusion rate with the consideration of non-Gaussianity;
3. $S={S}_{0}\exp \left[{\left(-{bDDC}\right)}^{\alpha }\right]$ for the SEM, where α reflects the diffusion non-Gaussianity, and DDC reflects the non-Gaussian diffusion rate;
4. $S={S}_{0}\exp [-D{{{{{{\rm{\mu }}}}}}}^{2\left(\beta -1\right)}{\left(\gamma {G}_{d}\delta \right)}^{2\beta }(\varDelta -\frac{2\beta -1}{2\beta +1}\delta )]$ for the FROC, where β reflects spatial fractional-order index, and μ is another spatial metric to preserve unites of mm^2^/s for D;
5. $S={S}_{0}{E}_{\alpha }[-{\left({bD}\right)}^{\beta }]$ for the CTRW, where α and β reflect the wait time and step length in each water molecular movement, and D reflects the diffusion rate, taking into account the temporal and spatial heterogeneity.

### Histogram analysis

On the ADC map and any one of the non-Gaussian diffusion metric maps, the volume of interest (VOI) was manually segmented around the whole tumor by two radiologists (A and B, respectively with 10 and 8 years of experience in MRI diagnosis, blinded to pathological results), using ITK-SNAP (v3.8.0, http://www.itksnap.org). T2-weighted, conventional diffusion-weighted (*b* = 800 sec/mm^2^), and DCE images were used as references for adjusting delineation, the necrosis regions (high T2-weighted, low diffusion-weighted, and low DCE signals) and tumor stalks (low diffusion-weighted signals) within VOIs were excluded. In cases with multiple tumors, the lesion with highest Vesical Imaging Reporting and Data System (VI-RADS) score was selected. Radiologist C (30 years of experience in MRI diagnosis) reviewed the VOIs delineated by the two radiologists and resolved controversies regarding lesion boundaries, necrotic regions, tumor stalks, and VI-RADS scores.

The 13 non-Gaussian diffusion metric maps of IVIM-f, IVIM-D, IVIM-D*, DKI-K, DKI-D, SEM-α, SEM-DDC, FROC-μ, FROC-β, FROC-D, CTRW-α, CTRW-β, and CTRW-D had identical dimensions, allowing the VOI to be directly copied between them without the need of registration. Then, employing FAE v0.5.6 (https://github.com/salan668/FAE), a tool developed within the open-resource framework pyradiomics, 18 types of histogram features (Appendix Table S3) were extracted from the ADC map and the 13 non-Gaussian diffusion metric maps, resulting in a total of 252 features per participant. The pipeline of image post-processing and histogram analysis is shown in Fig. 1B.

### VI-RADS scoring

Radiologists A, B and C have 5 years of experience in interpreting VI-RADS. Since DCE scanning was not performed on all participants, a bi-parametric VI-RADS based on T2-weighted and conventional diffusion-weighted images was used for lesions without DCE images, while a multi-parametric VI-RADS was used for lesions with DCE images. Referencing the standard scoring system [19, 20], the distributions of bi-parametric and multi-parametric VI-RADS scores are shown in Table 1.

**Table 1:** Clinical characteristics of participants in training and testing Cohorts

Characteristics	Training cohort (*n* = 267)	Testing cohort (*n* = 83)	*p* value
Age	67 (46–82)	65 (31–82)	0.329
Gender			0.293
Female	45 (16.9)	10 (12.0)	
Male	222 (83.1)	73 (88.0)	
Smoking			0.561
Yes	132 (49.4)	38 (45.8)	
No	135 (50.6)	45 (54.2)	
Number of lesions			0.249
Single	199 (74.5)	67 (80.7)	
Multiple	68 (25.5)	16 (19.3)	
Location of lesions			0.074
Trigone	31 (11.6)	16 (19.3)	
Others	236 (88.4)	67 (80.7)	
Diameter of lesions (cm)			0.338
Less than 3	178 (66.7)	60 (72.3)	
At least 3	89 (33.3)	23 (27.7)	
Pathologic T stage			0.539^a^
Ta	61 (22.8)	26 (31.3)	
T1	133 (49.8)	35 (42.2)	
T2	47 (17.6)	16 (19.3)	
T3	11 (4.2)	3 (3.6)	
T4	15 (5.6)	3 (3.6)	
Pathologic N stage			
N0	222 (83.1)	70 (84.3)	0.999^a^
N1	29 (10.9)	9 (10.8)	
N2	12 (4.5)	3 (3.6)	
N3	4 (1.5)	1 (1.3)	
Pathologic grade of NMIBC			0.481
Low	124 (63.9)	42 (68.7)	
High	70 (36.1)	19 (31.1)	
Multi-parametric VI-RADS			0.292^a^
1	11 (13.3)	2 (10.5)	
2	54 (65.1)	9 (47.4)	
3	6 (7.2)	4 (21.1)	
4	6 (7.2)	2 (10.5)	
5	6 (7.2)	2 (10.5)	
Bi-parametric VI-RADS			0.971^a^
1	21 (11.4)	8 (12.5)	
2	100 (54.3)	36 (56.3)	
3	36 (19.6)	10 (15.6)	
4	14 (7.6)	5 (7.8)	
5	13 (7.1)	5 (7.8)	

Unless otherwise specified, data out brackets represent numbers of patients, data in brackets represent percentages, analyzed by chi-square test

Data of Age out brackets represent medians, in brackets represent interquartile ranges, analyzed by Mann–Whitney *U* test

*MIBC* muscle-invasive bladder cancer, *NMIBC* non-muscle-invasive bladder cancer

^a^Fisher’s exact test

### Statistical analysis

Statistical analyses were conducted using R v4.2.1 (https://www.r-project.org) and Python v3.9.7 (https://www.python.org), with a two-sided *p*-value threshold set at less than 0.05. The Mann–Whitney *U* test and Chi-square test were employed for two-group comparisons of continuous data and categorical data, respectively.

In the training cohort, histogram features with an intraclass correlation coefficient (ICC, a two-way random effects model with two raters) below 0.80 were excluded to ensure robustness. Based on the groups of MIBC vs. NMIBC, and HG-NMIBC and LG-NMIBC, useful features of CTRW, DKI, FROC, IVIM, SEM, and DWI_conv_ were sequentially identified by least absolute shrinkage and selection operator (LASSO) combined with 10-fold cross-validation, to construct logistic regression (LR) models for each individual diffusion model. Significant features (*p* value of LR less than 0.05) from individual diffusion models were selected for correlation analysis. After excluding redundant features (the absolute value of Spearman coefficient > 0.70 and lower ICC), the remaining features were used to construct the combined diffusion model. The area under the receiver operating characteristic (ROC) curve (AUC) was the main index of model evaluation.

In the testing cohort, accuracy, sensitivity, and specificity under maximal Youden’s index were calculated to test the diagnostic performances of the models. The Delong method was performed to compare the differences in AUC values between the models. Decision curve analysis (DCA) was used to compare the clinical utility of the models by examining the net benefit across a range of threshold probabilities. The Hosmer-Lemeshow (H-L) test was employed to assess the fitting accuracy of the models.

## Results

### Participant characteristics

Overall, 350 consecutive eligible participants with urothelial carcinoma were included in this study. Among the 267 participants (222 men, 45 women; median age, 67 years [IQR: 46–82]) assigned to MR1, 73 (27.3%) were diagnosed with MIBC, and 194 (72.7%) with NMIBC. Within the NMIBC group, 70 (36.1%) were identified as HG, and 124 (63.9%) as LG. Among the 83 participants (73 men, 10 women; median age, 65 years [IQR: 31–82]) assigned to MR2, 22 (26.5%) participants were diagnosed with MIBC, and 61 (73.5%) participants were diagnosed with NMIBC. In the NMIBC group, 19 (31.1%) were identified as HG, and 42 (68.7%) as LG. The comparison of clinical and pathological characteristics of participants in MR1 and MR2 is shown in Table 1.

### Correlation of diffusion metrics with muscle invasion in bladder cancer

The intraclass correlation coefficient (ICC) values of 252 histogram features ranged from 0.648 to 0.998 (Appendix Table S4). Two features with ICCs below 0.80, specifically DKI-K-Kurtosis (ICC = 0.779) and SEM-α-Kurtosis (ICC = 0.614) were excluded.

In the training cohort, LASSO identified several useful metrics for classifying MIBC and NIMIBC, from each of the individual diffusion models (Table 2), the univariate analysis results of these metrics were documented in Appendix Table S5. CTRW-α-skewness, CTRW-D-mean, CTRW-D-skewness, DKI-D-mean, DKI-K-median, FROC-D-mean, FROC-D-skewness, IVIM-D-median, IVIM-D*-uniformity, SEM-DDC-skewness, DWI_conv_-mean, and DWI_conv_-skewness were significant relevant metrics in each individual diffusion model’s LR analysis, with all the *p* values less than 0.05. Among them, DKI-D-mean, DKI-K-median, FROC-D-mean, SEM-DDC-skewness, DWI_conv_-mean, and DWI_conv_-skewness, which had lower ICCs and strong correlations with other metrics, were excluded from the combined diffusion model LR analysis (Appendix Table S4).

**Table 2:** Diffusion models in diagnosing muscle invasion of bladder cancer

Model	Training cohort			Testing cohort			
	Coef	*p* value	AUC (95% CI)	AUC (95% CI)	ACC	Sen	Spe
CTRW			0.966 (0.946–0.986)	0.915 (0.775–0.932)	87%	91%	85%
Intercept	11.104	< 0.001					
α-skewness	0.654	0.003					
D-mean^a^	−0.007	< 0.001					
D-skewness	1.626	0.002					
DKI			0.839 (0.784–0.894)	0.806 (0.679–0.933)	80%	77%	80%
Intercept	−2.832	0.17					
D-mean^a^	−0.001	0.048					
D-skewness	0.857	0.119					
D-uniformity	0.3	0.956					
K-median	0.005	0.006					
FROC			0.850 (0.797–0.902)	0.843 (0.720–0.965)	90%	77%	95%
Intercept	1.181	0.409					
D-90P^a^	0	0.711					
D-mean^a^	0	0.034					
D-skewness	0.941	0.006					
μ-uniformity	1.87	0.448					
IVIM			0.840 (0.789–0.891)	0.838 (0.727–0.950)	80%	86%	77%
Intercept	0.394	0.705					
D-kurtosis	0.007	0.953					
D-median^a^	−0.003	0					
D-skewness	0.672	0.183					
D*-uniformity	2.644	0.037					
f-skewness	0.721	0.09					
SEM			0.839 (0.783–0.895)	0.781 (0.645–0.907)	81%	59%	89%
Intercept	0.475	0.638					
α-energy	0	0.756					
α-TE	0	0.753					
DDC-mean^a^	−0.001	0.42					
DDC-median^a^	0	0.678					
DDC-skewness	0.758	0.019					
DWI_conv_			0.848 (0.798–0.899)	0.805 (0.645–0.907)	78%	77%	79%
Intercept	1.337	0.286					
ADC-90P^a^	0	0.632					
ADC-mean^a^	−0.002	0.034					
ADC-skewness	1.25	0					
Combined			0.968 (0.947–0.988)	0.885 (0.790–0.980)	88%	82%	90%
Intercept	9.282	0					
CTRW-α-skewness	0.788	0.002					
CTRW-D-mean^a^	−0.008	0					
CTRW-D-skewness	0.84	0.191					
FROC-D-skewness	1.189	0.066					
IVIM-D-median^a^	0.003	0.167					
IVIM-D*uniformity	0.698	0.726					

*CTRW* continuous time random walk, *DKI* diffusion kurtosis imaging, *FROC* fractional-order calculus, *IVIM* intravoxel incoherent motion, *SEM* stretched exponential model, *DWI*_*conv*_ conventional diffusion-weighted imaging, *ADC* apparent diffusion coefficient, *90* *P* 90th percentile, *TE* total energy, *CI* confidence interval, *ACC* accuracy, *Sen* sensitivity, *Spe* specificity

^a^×10^−3^ μm^2^/ms

**Fig. 2:**
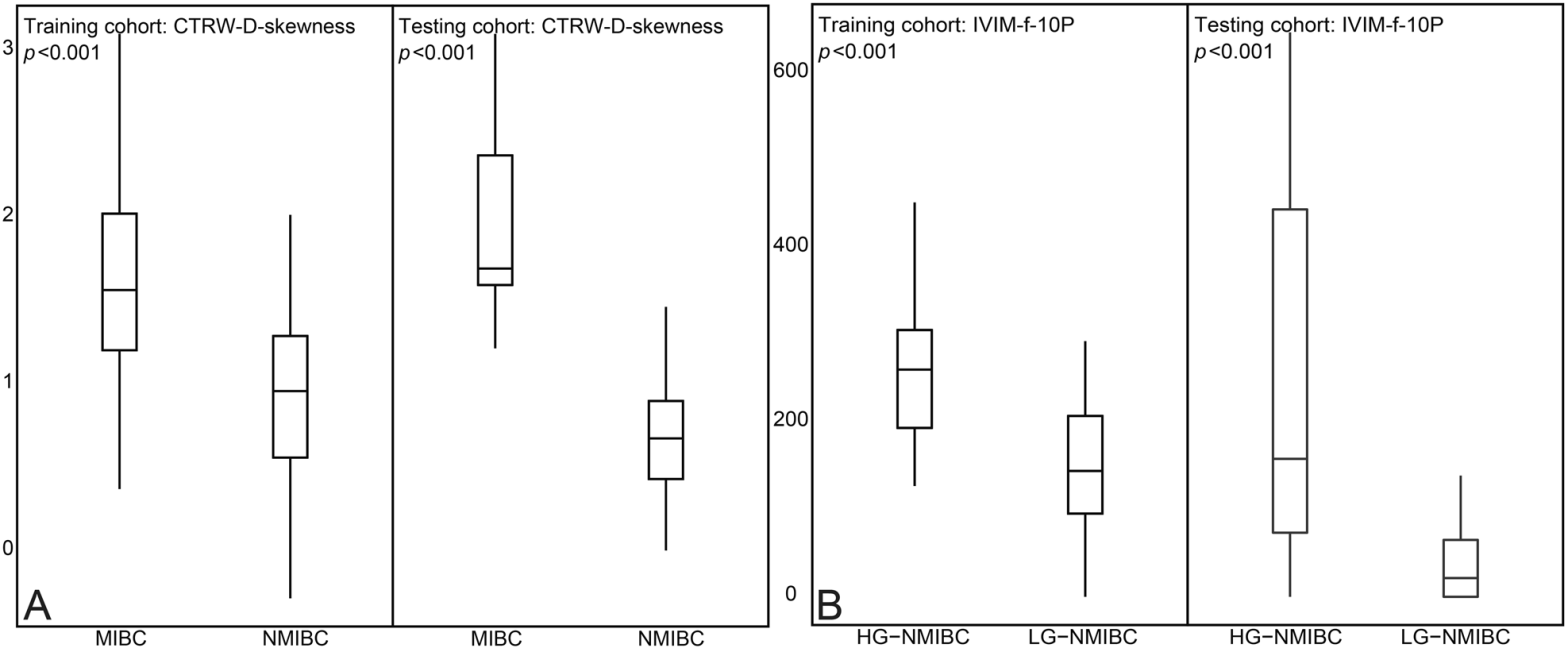
Distributions of the most useful metrics for assessing (**A**) muscle invasion of bladder cancer and (**B**) histological grade of non-muscle-invasive bladder cancer in training and testing cohorts. CTRW, continuous time random walk; IVIM, intravoxel incoherent motion

CTRW-D-skewness, DKI-D-skewness, FROC-μ-uniformity, IVIM-D*-uniformity, SEM-DDC-skewness, and DWI_conv_-ADC-skewness were the representative metrics in each individual diffusion model, with the highest LR coefficient. In the training cohort, the six representative metrics were significantly higher in MIBC than in NMIBC, with all the *p* values less than 0.001. In the testing cohort, except for IVIM-D*-uniformity (*p* = 0.093), the other five representative metrics also significantly higher in MIBC than in NMIBC, with all the *p* values less than 0.001 (Appendix Table S5). The distributions of CTRW-D-skewness, the most useful metric for diagnosing muscle invasion of BCa, in both the training and testing cohorts are shown in Fig. 2A.

### Diagnostic performance of diffusion metrics for muscle invasion in bladder cancer

**Fig. 3:**
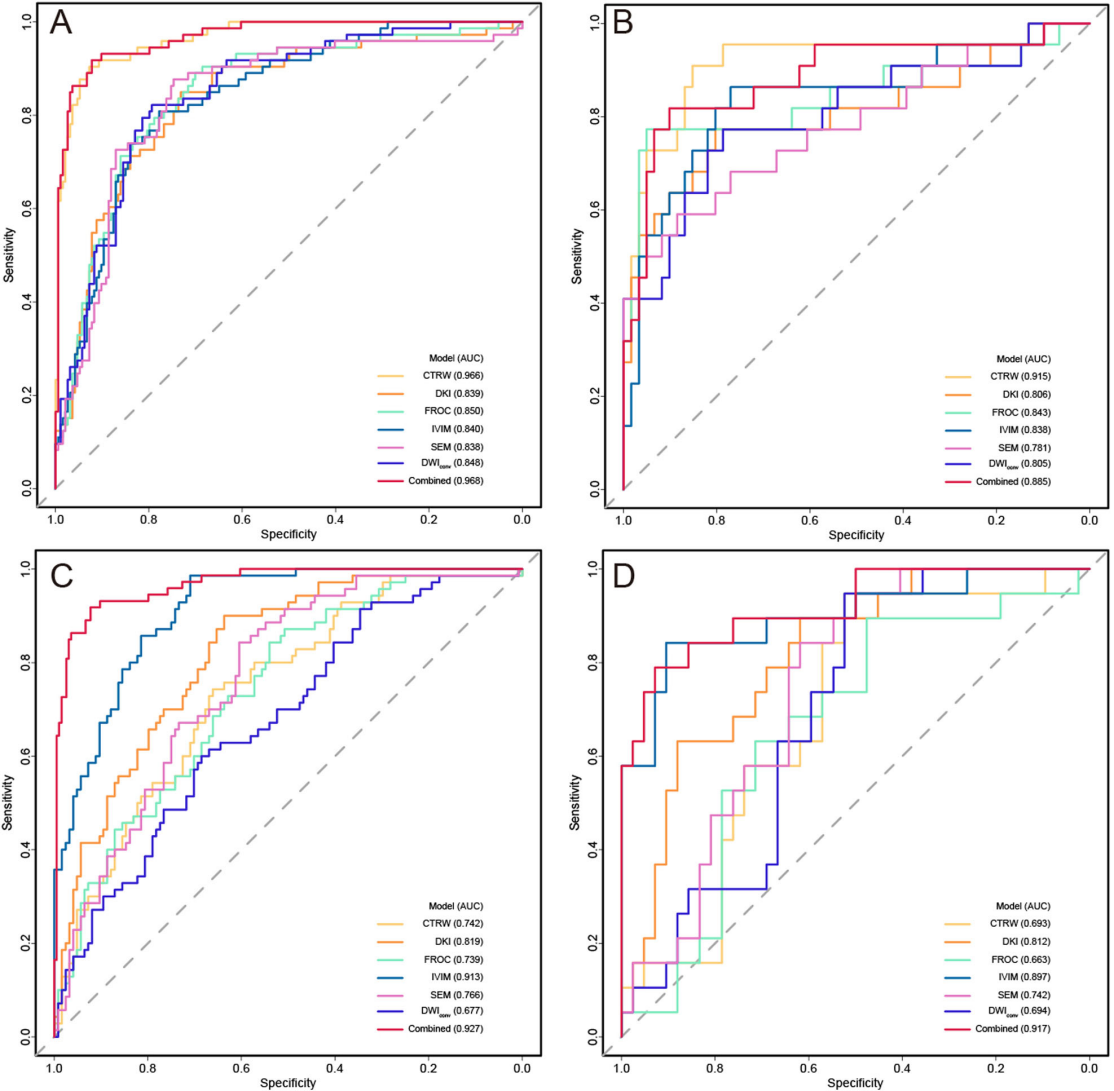
Receiver operating character (ROC) curves for diffusion models. **A**, **B** ROC curves for the diagnosis of muscle invasion in the (**A**) training and (**B**) testing cohorts, respectively. **C**, **D** ROC curves for the diagnosis of histological grade in the (**C**) training and (**D**) testing cohorts. respectively. AUC, area under the receiver operating characteristic curves; CI, confidence interval; CTRW, continuous time random walk; DKI, diffusion kurtosis imaging; FROC, fractional-order calculus; IVIM, intravoxel incoherent motion; SEM, stretched exponential model; DWI_conv_, conventional diffusion-weighted imaging

**Fig. 4:**
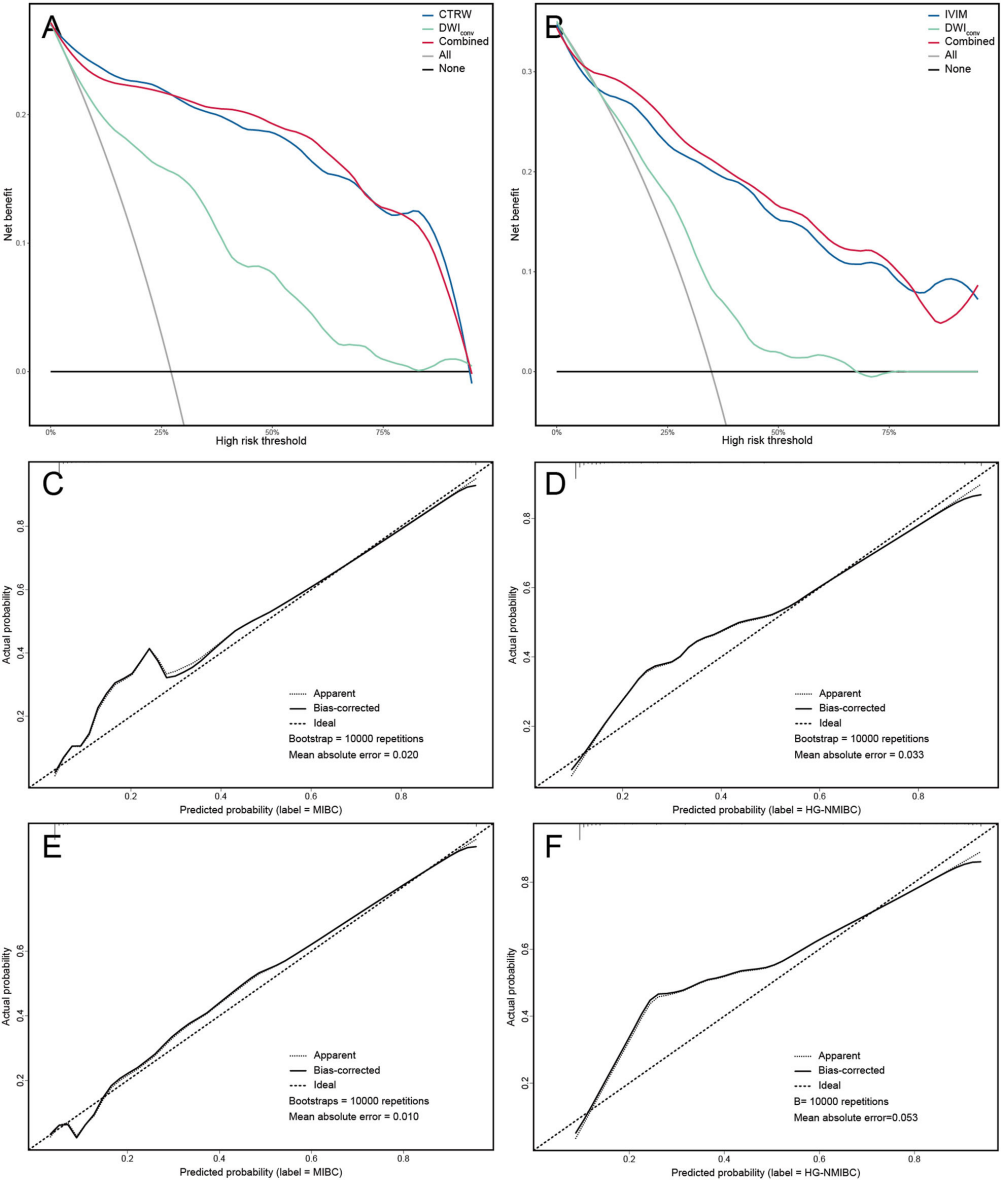
Decision curve analysis (DCA) and calibration curves. Comparison of net benefit among best individual non-Gaussian diffusion models, conventional diffusion-weighted imaging (DWI_conv_) models, and combined diffusion models for diagnosing (**A**) muscle invasion and (**B**) histological grade. Calibration curves of (**C**, **D**) best individual non-Gaussian diffusion models and (**E**, **F**) combined diffusion models for diagnosing (**C**, **E**) muscle invasion and (**D**, **F**) histological grade. Continuous time random walk (CTRW) and intravoxel incoherent motion (IVIM) are the best non-Gaussian individual diffusion models with the highest area under the receiver operating characteristic curve for diagnosing muscle invasion and histological grade, respectively.

LR analysis showed that the CTRW, DKI, FROC, IVIM, SEM, DWI_conv_, and the combined diffusion model performed AUCs of 0.966, 0.839, 0.850, 0.840, 0.839, 0.848, and 0.968 for diagnosing muscle invasion in the training cohort, respectively. Correspondingly, they performed AUCs of 0.915, 0.806, 0.843, 0.838, 0.781, 0.805, and 0.885 respectively in the testing cohort (Table 2, Fig. 3). In the comparison of AUCs in the testing cohort (Table 3), the AUC of CTRW was significantly higher than that of DWI_conv_ (*p* = 0.014), and similar to the combined diffusion model (*p* = 0.076). CTRW was a highly sensitive non-Gaussian diffusion model for diagnosing muscle invasion, and it reached the highest sensitivity of 91% among all diffusion models. The DCA (Fig. 4) showed that across all risk threshold probabilities, the clinical net benefit of the CTRW was similar to that of the combined diffusion model, and both significantly superior to the DWI_conv_. In addition, the calibration curve of the CTRW also demonstrated an acceptable fitting condition, with the mean absolute error (MAE) value of 0.020 based on 10,000 bootstrap repetitions. Meanwhile, the combined model showed an MAE of 0.010.

**Table 3:** Comparison of diffusion models for determining muscle invasion and grade of bladder cancer

	CTRW	DKI	FROC	IVIM	SEM	DWI_conv_	Combined
CTRW	…	0.047^a^	0.087^a^	0.107^a^	0.003^a^	0.014^a^	0.076^a^
DKI	0.028^b^	…	0.327^a^	0.544^a^	0.554^a^	0.964^a^	0.111^a^
FROC	0.468^b^	0.005^b^	…	0.890^a^	0.054^a^	0.117^a^	0.179^a^
IVIM	0.020^b^	0.214^b^	0.007^b^	…	0.191^a^	0.400^a^	0.237^a^
SEM	0.211^b^	0.149^b^	0.052^b^	0.047^b^	…	0.428^a^	0.033^a^
DWI_conv_	0.988^b^	0.150^b^	0.760^b^	0.020^b^	0.542^b^	…	0.008^a^
Combined	0.002^b^	0.021^b^	0.000^b^	0.650^b^	0.003^b^	0.004^b^	…

*CTRW* continuous time random walk, *DKI* diffusion kurtosis imaging, *FROC* fractional-order calculus, *IVIM* intravoxel incoherent motion, *SEM* stretched exponential model, *DWI* diffusion-weighted imaging

^a^The *p* values obtained from Delong’s test for the diffusion models applied in predicting muscle invasion

^b^The *p* values obtained from Delong’s test for the diffusion models applied in assessing histological grade

### Correlation of diffusion metrics with histological grade in non-muscle-invasive bladder cancer

For the assessment of the histological grade of NMIBC, several useful metrics were identified by LASSO from each individual diffusion model in the training cohort (Table 4), the comparisons of these metrics between HG and LG were noted in Appendix Table S6. Significant relevant metrics in the individual diffusion models include CTRW-β-skewness, CTRW-D-10P, DKI-K-mean, DKI-K-median, FROC-β-median, FROC-β-skewness, IVIM-D-median, IVIM-f-10P, SEM-α-median, SEM-α-skewness, and SEM-DDC-10P, with all the *p* values of LR analysis less than 0.05. LR analysis of DWI_conv_ showed that no metrics had a significant correlation to the histological grade of NMIBC, with the *p* values of 0.098 (DWI_conv_-ADC-10P), 0.844 (DWI_conv_-ADC-median), and 0.307 (DWI_conv_-ADC-skewness). Among the significant metrics, CTRW-D-10P, DKI-K-median, FROC-β-skewness, IVIM-D-median, SEM-α-median, and SEM-α-skewness had strong correlations with other metrics and had weaker consistencies were excluded for combined diffusion model LR analysis.

**Table 4:** Diffusion models in assessing histological grade of non-muscle-invasive bladder cancer

Model	Training cohort			Testing cohort			
	Coef	*p* value	AUC (95% CI)	AUC (95% CI)	ACC	Sen	Spe
CTRW			0.742 (0.672–0.813)	0.693 (0.556–0.830)	66%	95%	52%
Intercept	3.998	0.042					
β-median	−0.003	0.306					
β-skewness	0.907	0.04					
D-10P^a^	−0.002	0					
DKI			0.819 (0.759–0.879)	0.812 (0.701–0.923)	70%	90%	62%
Intercept	−7.257	0					
K-mean	0.006	0.031					
K-median	0.005	0.08					
FROC			0.739 (0.669–0.810)	0.663 (0.516–0.809)	61%	90%	48%
Intercept	6.218	0.002					
β-median	−0.008	0.002					
β-skewness	0.724	0.019					
IVIM			0.913 (0.875–0.951)	0.897 (0.801–0.993)	89%	84%	90%
Intercept	0.217	0.844					
D-median^a^	−0.005	0					
f-10P	0.022	0					
SEM			0.766 (0.699–0.833)	0.742 (0.620–0.864)	69%	84%	62%
Intercept	6.593	0.001					
α-median	−0.007	0.008					
α-skewness	0.855	0.004					
DDC-10P^a^	−0.001	0.007					
DWI_conv_			0.677 (0.600–0.754)	0.694 (0.563–0.826)	66%	95%	52%
Intercept	0.591	0.614					
ADC-10P^a^	−0.002	0.098					
ADC-median^a^	0	0.844					
ADC-skewness^a^	0.412	0.307					
Combined			0.927 (0.892–0.961)	0.917 (0.839–0.996)	89%	79%	93%
Intercept	−3.613	0.014					
CTRW-β-skewness	0.888	0.146					
DKI-K-mean	0.01	0.003					
FROC-β-median	0.006	0.282					
IVIM-f-10P	0.024	0					
SEM-DDC-10P^a^	−0.002	0.067					

*CTRW* continuous time random walk, *DKI* diffusion kurtosis imaging, *FROC* fractional-order calculus, *IVIM* intravoxel incoherent motion, *SEM* stretched exponential model, *DWI*_*conv*_ conventional diffusion-weighted imaging, *ADC* apparent diffusion coefficient, *10P* 10th percentile, *CI* confidence interval, *ACC* accuracy, *Sen* sensitivity, *Spe* specificity

^a^×10^−3^ μm^2^/ms

In LR analysis of the single diffusion models, CTRW-β-skewness, DKI-K-mean, FROC-β-skewness, IVIM-f-10P, SEM-α-skewness, DWI_conv_-ADC-skewness were representative metrics with the highest coefficient. The comparisons, based on the Mann–Whitney *U* test, showed that, in the training cohort, all representative metrics of HG-NMIBC were significantly higher than those of LG-NMIBC, with all the *p* values less than 0.05. In the testing cohort, DKI-K-mean, IVIM-f-10P, and DWI_conv_-ADC-skewness of HG-NMIBC were significantly higher than those of LG-NMIBC, with all the *p* values less than 0.05 (Appendix Table S6). The distributions of IVIM-f-10P, the most useful metric for assessing the histological grade of NMIBC, in both training and testing cohorts, are shown in Fig. 2B.

### Diagnostic performance of diffusion metrics for histological grade in non-muscle-invasive bladder cancer

In the training cohort, the LR analysis of CTRW, DKI, FROC, IVIM, SEM, DWI_conv_, and the combined diffusion model performed the AUCs of 0.742, 0.819, 0.739, 0.913, 0.766, 0.677, and 0.927 respectively for assessing the histological grade of NMIBC (Table 4). In the testing cohort, the AUCs of these models were 0.693, 0.812, 0.663, 0.897, 0.742, 0.694, and 0.917, respectively. The comparison of AUCs in the testing cohort showed that IVIM performed better than DWI_conv_ (*p* = 0.020), and similarly to the combined diffusion model (*p* = 0.650). IVIM and the combined diffusion model both achieved the highest testing accuracy of 89%. However, the former exhibited a higher sensitivity of 84%, while the latter demonstrated a higher specificity of 93%. Across most risk threshold probabilities, the clinical benefit of the combined diffusion model was slightly higher than that of the IVIM, with both significantly outperforming the DWI_conv_ (Fig. 4). However, IVIM demonstrated a higher goodness-of-fit compared to the combined diffusion model. Based on the 10,000 bootstrap repetitions, IVIM achieved a lower MAE of 0.033, compared to 0.053 of the combined diffusion model.

## Discussion

This research is characterized by several notable strengths. First, we utilized the largest dataset to date to validate the performance of non-Gaussian diffusion models for diagnosing muscle invasion and histological grade of BCa. Second, we employed two MRI scanners for image acquisition, with one scanner’s data serving as an independent testing cohort, ensuring the robustness of our results. Third, all non-Gaussian diffusion models were obtained simultaneously through post-processing of a single multi-*b*-value scan, eliminating the need for repetitive scans. Furthermore, through comparison, we found that CTRW and IVIM were potential individual non-Gaussian models for BCa diagnosis. In diagnosing muscle invasion, the testing AUC of CTRW (0.915) was the highest and significantly higher than that of DWI_conv_ (0.805, *p* = 0.014). In diagnosing the histological grade of NMIBC, the testing AUC of IVIM (0.897) was similar to that of the combined diffusion model (0.917, *p* = 0.650), and both significantly higher than the testing AUC of DWI_conv_ (both the *p* values below 0.05). With similar diagnostic performance to the combined diffusion models, individual diffusion models are structurally simpler and easier to apply in clinical practice.

Accurate preoperative assessment of muscle invasion and histological grade of BCa is crucial for therapeutic decisions [21, 22]. VI-RADS is a sensible tool for diagnosing muscle invasion of BCa, but the risk interpretation of lesions with a score of 3 remains ambiguous, and the diagnostic cutoff scores are still controversial [19, 20]. Moreover, although VI-RADS aims to standardize the interpretation of MRI in BCa, its morphology-based assessments still tend to be influenced by the subjective experience of readers. In contrast, quantitative assessment results based on the microstructural characteristics of the tumor are more objective and stable. CTRW, the best quantitative non-Gaussian diffusion model for diagnosing muscle invasion of BCa in this study, characterizes the nonlinear diffusion of water molecules, which are “trapped” or “released” by tumor microstructures, using the temporal metric α and spatial metric β [12, 14, 15, 23]. D-skewness, α-skewness and D-mean were useful metrics in our CTRW model. MIBC, characterized by deep invasion into the bladder wall, exhibits higher cellular density, leading to a lower CTRW-D-mean value (Appendix Table S5) compared to NMIBC [24]. And the heterogeneity of cell proliferative cycle stages, morphology, size, arrangement, and vascular distribution within MIBC, contributes more variable waiting times and step lengths for particle diffusion [23]. This results in higher CTRW-α-skewness and CTRW-D-skewness values (Appendix Table S5).

Compared to CTRW, IVIM is a typical compartmentalized non-Gaussian diffusion model that divides tumors into microvascular perfusion compartments and intercellular diffusion compartments for analysis [25]. This allows for the separate assessment of hemodynamic properties (metrics f and D*) and actual interstitial diffusion (metric D). In this study, IVIM emerged as the most useful model for assessing histological of NMIBC, with its most significant metric, f-10P, indicating that perfusion constitutes a key biological difference between HG and LG tumors. Unlike LG-NMIBC, HG-NMIBC exhibits a higher cell proliferation, leading to an increased demand for perfusion [26, 27]. Thus, the IVIM-f-10P, representing the 10th percentile of vascular volume fraction in tumor, was significantly higher in HG-NMIBC than in LG-NMIBC. The higher proliferation level also results in congested interstitial spaces, reducing D-mean in HG-NMIBC (Appendix Table S6) [28–30].

Notably, among six representative metrics in each task, four of them were skewness metrics. In the CTRW model, two of the three were skewness metrics. Skewness, describing the asymmetry of data distribution, better captures the heterogeneity of diffusion behavior in tumors, as opposed to the mean, showing stronger resistance to outliers [31]. Thus, skewness better describes the heterogeneity of diffusion behavior in tumors. In the study of Cui et al [15], the AUCs for diagnosing muscle invasion based on FROC (β-mean, μ-mean, and D-mean) and DWI_conv_ (ADC-mean) models were only 0.782 and 0.730. While through incorporating skewness metrics, the AUCs of FROC and DWI_conv_ models in our study improved to 0.843 and 0.805.

The current diagnostic process for BCa relies largely on TURBT. However, a single TURBT often presents insufficient staging and grading, and its extent and depth of resection are determined by the experience of urologists [3–5]. Repeated invasive diagnosis caused mucosal damage, infections, and patient substantial distress, and waste of medical resources [32–34]. Our results demonstrated the non-Gaussian diffusion models of CTRW and IVIM possess acceptable accuracy and strong robustness in assessing muscle invasion and histological grade. This finding could advance the application of non-Gaussian diffusion models in clinical practice, particularly amplifying the standing of MRI in current diagnostic procedures for BCa and reducing the dependence on TURBT.

Our study had limitations. First, both scanners originated from a single institution, suggesting that further multicenter studies are needed to validate the reproducibility of these results. Second, excluding lesions with a maximum diameter of less than 5 mm or imaged in fewer than 3 slices may introduce selection bias. Third, the long acquisition time required for multi-*b*-value diffusion-weighted scanning might cause discomfort to patients with low tolerance. In addition, the time cost of image post-processing needs to be further shortened. However, clinical practice does not necessitate the use of all non-Gaussian diffusion models. By focusing solely on the CTRW and IVIM models, there is no need for scanning with 11 b-values, which could significantly reduce both scanning and post-processing times.

**Fig. 5:**
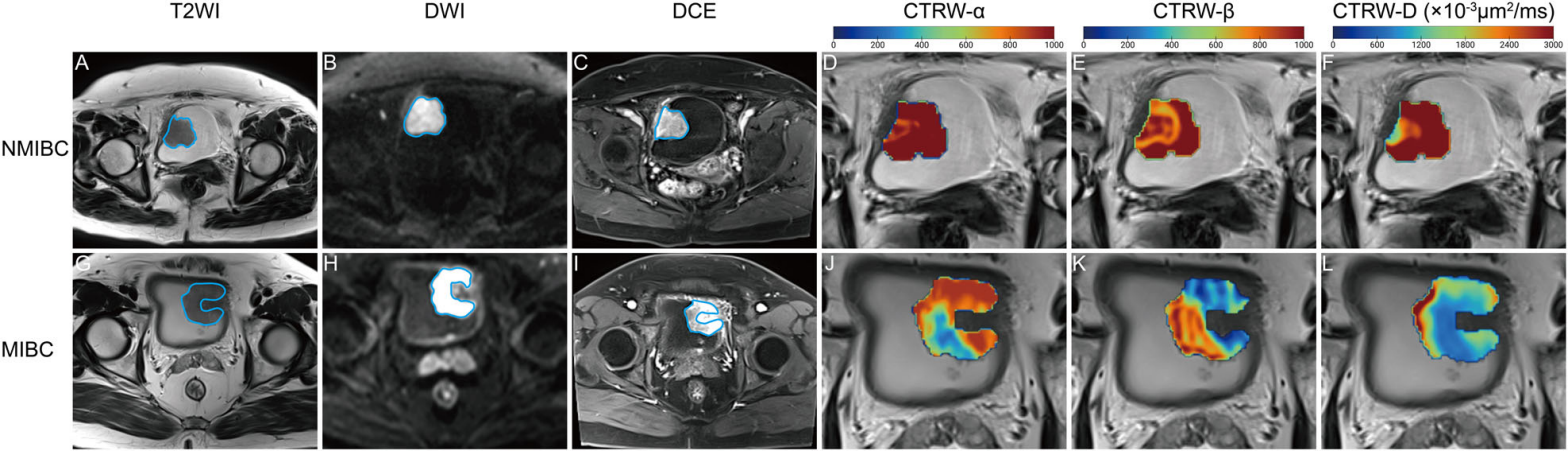
T2-weighted (T2WI), conventional diffusion-weighted (DWI), and dynamic contrast-enhanced (DCE) images, and CTRW metric maps from (**A**–**F**) a 69-year-old male with non-muscle-invasive bladder cancer (NMIBC) and (**G**–**L**) a 60-year-old male with muscle-invasive bladder cancer (MIBC). **A**, **G** T2WI, (**B**, **H**) DWI, and (**C**, **I**) DCE, along with (**D**, **J**) CTRW-α, (**E**, **K**) CTRW-β, and (**F**, **L**) CTRW-D metric maps integrated into the corresponding T2WI within the tumors. In both patients, the Vesical Imaging Reporting and Data System assigned a score of 3. Additionally, all metric maps indicated that the lesion in the MIBC appeared cooler and exhibited more variability in color, suggesting a higher degree of diffusion limitation and greater heterogeneity, both temporally and spatially, within the lesion

**Fig. 6:**
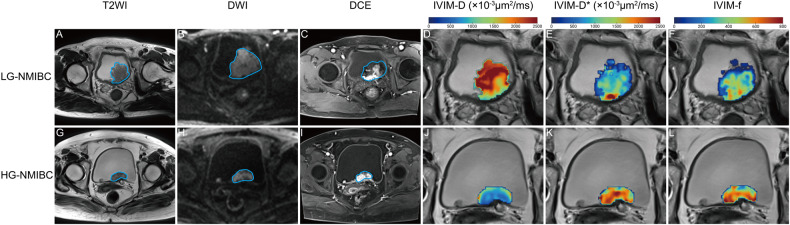
T2-weighted (T2WI), conventional diffusion-weighted (DWI), and dynamic contrast-enhanced (DCE) images, and CTRW metric maps from (**A**–**F**) a 60-year-old male with low-grade non-muscle-invasive bladder cancer (LG-NMIBC) and (**G**–**L**) a 63-year-old male with high-grade non-muscle-invasive bladder cancer (HG-NMIBC). **A**, **G** T2WI, (**B**, **H**) DWI, and (**C**, **I**) DCE, along with (**D**, **J**) IVIM-D, (**E**, **K**) IVIM-D*, and (**F**, **L**) IVIM-f metric maps integrated into the corresponding T2WI within the tumors. In both patients, the Vesical Imaging Reporting and Data System assigned a score of 3. The value of IVIM-D in HG tumor was significantly lower, indicating the higher cellular density. Additionally, the color representation of IVIM-D* and IVIM-f in the HG tumor appeared hotter and exhibited more variability, suggesting a higher degree of perfusion and greater heterogeneity within the lesion

## Conclusions

CTRW and IVIM are two potential non-Gaussian diffusion models to assess muscle invasion and histological grade for BCa, respectively. Compared with the DWI_conv_, the new models improved the accuracy and sensitivity, ultimately, contributing to improved outcomes in the management of BCa.

### Supplementary information

Electronic Supplementary Material

## Data Availability

The data and materials used in this research are available upon request. We are committed to promoting transparency and reproducibility in our research. For access to the data and materials, please contact Xiaochun Wang at 2010xiaochun@163.com. We will provide the requested data and materials promptly to qualified researchers for the purpose of academic and scientific inquiry.

## Abbreviations

ADC: Apparent diffusion coefficient
AUC: Area under the ROC curve
BCa: Bladder cancer
CTRW: Continuous time random walk
DCA: Decision curve analysis
DKI: Diffusion kurtosis imaging
DWI_conv_: Conventional diffusion-weighted imaging
FROC: Fractional-order calculus
HG: High-grade
ICC: Intraclass correlation coefficient
IVIM: Intravoxel incoherent motion
LASSO: Least absolute shrinkage and selection operator
LG: Low-grade
LR: Logistic regression
MAE: Mean absolute error
MIBC: Muscle-invasive bladder cancer
NMIBC: Non-muscle-invasive bladder cancer
ROC: Receiver operating characteristic
SEM: Stretched exponential model
TURBT: Transurethral resection of bladder tumor
