# Supplementary material for: Non-Gaussian diffusion metrics with whole-tumor histogram analysis for bladder cancer diagnosis: muscle invasion and histological grade
Source: Insights Imaging. 2024 Jun 9;15:138. doi: 10.1186/s13244-024-01701-z (PMC11162990; doi:10.1186/s13244-024-01701-z)
Supplement: Supplementary file 1 — Electronic Supplementary Material [file 13244_2024_1701_MOESM1_ESM.pdf]

Non-Gaussian Diffusion Metrics with Whole-tumor Histogram Analysis for Bladder Cancer Diagnosis: Muscle Invasion and Histological Grade

ELECTRONIC SUPPLEMENTARY MATERIAL

Appendix E1 Specific parameters for imaging acquisition

Coil information: 32-channel spine coil or 18-channel body coil.

Multi-b-value information: 0, 50, 100, 150, 200, 500, 800, 1000, 1500, 2000, and 2500 sec/mm2.

Table S1 Sequences and acquisition parameters of MR1

Sequence	Planes	Fat Suppression	TR/TE (msec)	Slice Thickness/ gap (mm)	Flip Angle	FOV (mm^2)	Matrix	Sections
T1WI: TSE	Axial	-	550/10	6/7.2	150°	370×370	640×640	19
T2WI: TSE	Axial	-	4170/106	6/7.2	153°	370×370	384×384	19
	Axial	SPAIR	5900/106	6/7.2	150°	370×370	384×384	19
	Sagittal	SPAIR	3200/88	4/5.2	160°	260×260	384×384	20
	Coronal	SPAIR	3540/88	5/6.5	160°	320×320	320×320	20
DWI: EPI	Axial	Fat Sat	4100/65	6/7.2	90°	280×245	224×296	19
DCE: VIBE	Axial	SPAIR	3.2/1.2	3/3	9°	360×281	320×350	48
	Sagittal	SPAIR	3.1/1.2	3/3	9°	262×350	240×320	44
	Coronal	Fast Fat Sat	4.2/2.0	3.5/3.5	9°	320×320	320×320	36
Multi-b-value DWI: Slice-Specific Dynamic Shimming EPI	Axial	SPAIR	4400/74	4/4.8	90°	277×370	192×256	20

TR, repetition time; TE, echo time; FOV, field of view; T1WI, T1-weighted imaging; TSE, turbo spin echo, SPAIR, spectral attenuated inversion recovery; DWI, diffusion-weighted imaging; EPI, echo planar imaging; fat sat, fat saturated; VIBE, volumetric interpolated breath-hold examination

Table S2 Sequences and acquisition parameters of MR2

Sequence	Planes	Fat Suppression	TR/TE (msec)	Slice Thickness/ gap (mm)	Flip Angle	FOV (mm^2)	Matrix	Sections
T1WI: TSE	Axial	-	550/10	6/7.2	150°	370×370	640×640	19
T2WI: TSE	Axial	-	4170/106	6/7.2	149°	370×370	384×384	19
	Axial	SPAIR	5900/106	6/7.2	120°	370×370	384×384	19
	Sagittal	SPAIR	3200/88	4.5/5.9	160°	260×260	384×384	20
	Coronal	SPAIR	3540/88	5/6.5	160°	320×320	320×320	20
DWI: EPI	Axial	Fat Sat	4000/76	6/7.2	90°	280×245	224×296	19
DCE: VIBE	Axial	SPAIR	3.2/1.2	3/3	9°	360×281	320×250	48
	Sagittal	SPAIR	3.1/1.2	3/3	9°	262×350	240×320	40
	Coronal	Fast Fat Sat	4.2/2.0	3.5/3.5	9°	320×320	320×320	36
Multi-b-value DWI: SMS-EPI	Axial	SPAIR	3000/70	4/4.8	90°	277×370	180×240	20

TR, repetition time; TE, echo time; FOV, field of view; T1WI, T1-weighted imaging; TSE, turbo spin echo; SPAIR, spectral attenuated inversion recovery; DWI, diffusion-weighted imaging; EPI, echo planar imaging; fat sat, fat saturated; VIBE, volumetric interpolated breath-hold examination; SMS, simultaneous multi slice

Appendix E2 Histogram features exclusion and selection

From each diffusion metric map, 18 kinds of histogram features were extracted. From maps of apparent diffusion coefficient and 13 non-Gaussian diffusion metrics, a total of 252 histogram features were extracted. The 18 kinds of histogram are displayed in **Table S3**.

Table S3 Types of histogram features

Features		
10th percentile (10P)	90th percentile (90P)	energy
entropy	interquartile range (IR)	kurtosis
maximus	mean absolute deviation (MAD)	mean
median	minimum	range
robust mean absolute deviation (RMAD)	root mean squared (RMS)	skewness
total energy (TE)	uniformity	variance

Table S4 The intraclass correlation coefficient of all histogram features

Feature	ICC	Feature	ICC	Feature	ICC	Feature	ICC	Feature	ICC
CTRW_α_10P	0.966	CTRW_D_Maximum	0.947	DKI_D_RMAD	0.952	FROC_μ_10P	0.961	IVIM_D*_Maximum	0.955
CTRW_α_90P	0.939	CTRW_D_MAD	0.927	DKI_D_RMS	0.986	FROC_μ_90P	0.969	IVIM_D*_MAD	0.979
CTRW_α_Energy	0.997	CTRW_D_Mean	0.996	DKI_D_Skewness	0.888	FROC_μ_Energy	0.997	IVIM_D*_Mean	0.972
CTRW_α_Entropy	0.919	CTRW_D_Median	0.975	DKI_D_TE	0.999	FROC_μ_Entropy	0.974	IVIM_D*_Median	0.966
CTRW_α_IR	0.976	CTRW_D_Minimum	0.946	DKI_D_Uniformity	0.931	FROC_μ_IR	0.974	IVIM_D*_Minimum	0.959
CTRW_α_Kurtosis	0.981	CTRW_D_Range	0.952	DKI_D_Variance	0.939	FROC_μ_Kurtosis	0.955	IVIM_D*_Range	0.956
CTRW_α_Maximum	0.895	CTRW_D_RMAD	0.918	FROC_β_10P	0.964	FROC_μ_Maximum	0.943	IVIM_D*_RMAD	0.977
CTRW_α_MAD	0.963	CTRW_D_RMS	0.960	FROC_β_90P	0.958	FROC_μ_MAD	0.967	IVIM_D*_RMS	0.977
CTRW_α_Mean	0.969	CTRW_D_Skewness	0.987	FROC_β_Energy	0.997	FROC_μ_Mean	0.975	IVIM_D*_Skewness	0.889
CTRW_α_Median	0.968	CTRW_D_TE	0.983	FROC_β_Entropy	0.909	FROC_μ_Median	0.979	IVIM_D*_TE	0.995
CTRW_α_Minimum	0.874	CTRW_D_Uniformity	0.967	FROC_β_IR	0.946	FROC_μ_Minimum	0.853	IVIM_D*_Uniformity	0.958
CTRW_α_Range	0.885	CTRW_D_Variance	0.924	FROC_β_Kurtosis	0.847	FROC_μ_Range	0.935	IVIM_D*_Variance	0.984
CTRW_α_RMAD	0.972	DKI_D_10P	0.986	FROC_β_Maximum	0.939	FROC_μ_RMAD	0.972	IVIM_f_10P	0.971
CTRW_α_RMS	0.972	DKI_D_90P	0.947	FROC_β_MAD	0.929	FROC_μ_RMS	0.976	IVIM_f_90P	0.959
CTRW_α_Skewness	0.985	DKI_D_Energy	0.983	FROC_β_Mean	0.978	FROC_μ_Skewness	0.904	IVIM_f_Energy	0.992
CTRW_α_TE	0.997	DKI_D_Entropy	0.958	FROC_β_Median	0.975	FROC_μ_TE	0.997	IVIM_f_Entropy	0.944
CTRW_α_Uniformity	0.913	DKI_D_IR	0.934	FROC_β_Minimum	0.977	FROC_μ_Uniformity	0.978	IVIM_f_IR	0.942
CTRW_α_Variance	0.925	DKI_D_Kurtosis	0.928	FROC_β_Range	0.900	FROC_μ_Variance	0.958	IVIM_f_Kurtosis	0.913
CTRW_β_10P	0.967	DKI_D_Maximum	0.950	FROC_β_RMAD	0.944	IVIM_D_10P	0.979	IVIM_f_Maximum	0.932
CTRW_β_90P	0.950	DKI_D_MAD	0.941	FROC_β_RMS	0.978	IVIM_D_90P	0.961	IVIM_f_MAD	0.949
CTRW_β_Energy	0.998	DKI_D_Mean	0.970	FROC_β_Skewness	0.900	IVIM_D_Energy	0.988	IVIM_f_Mean	0.973
CTRW_β_Entropy	0.923	DKI_D_Median	0.975	FROC_β_TE	0.997	IVIM_D_Entropy	0.966	IVIM_f_Median	0.971
CTRW_β_IR	0.953	DKI_D_Minimum	0.950	FROC_β_Uniformity	0.921	IVIM_D_IR	0.927	IVIM_f_Minimum	0.964
CTRW_β_Kurtosis	0.903	DKI_D_Range	0.956	FROC_β_Variance	0.900	IVIM_D_Kurtosis	0.944	IVIM_f_Range	0.948
CTRW_β_Maximum	0.945	DKI_D_RMAD	0.935	FROC_D_10P	0.991	IVIM_D_Maximum	0.930	IVIM_f_RMAD	0.944
CTRW_β_MAD	0.941	DKI_D_RMS	0.961	FROC_D_90P	0.932	IVIM_D_MAD	0.923	IVIM_f_RMS	0.970
CTRW_β_Mean	0.973	DKI_D_Skewness	0.954	FROC_D_Energy	0.981	IVIM_D_Mean	0.983	IVIM_f_Skewness	0.949
CTRW_β_Median	0.969	DKI_D_TE	0.983	FROC_D_Entropy	0.963	IVIM_D_Median	0.972	IVIM_f_TE	0.992
CTRW_β_Minimum	0.890	DKI_D_Uniformity	0.966	FROC_D_IR	0.903	IVIM_D_Minimum	0.913	IVIM_f_Uniformity	0.933
CTRW_β_Range	0.915	DKI_D_Variance	0.934	FROC_D_Kurtosis	0.915	IVIM_D_Range	0.930	IVIM_f_Variance	0.981
CTRW_β_RMAD	0.952	DKI_K_10P	0.969	FROC_D_Maximum	0.916	IVIM_D_RMAD	0.924	SEM_α_10P	0.959
CTRW_β_RMS	0.972	DKI_K_90P	0.986	FROC_D_MAD	0.903	IVIM_D_RMS	0.980	SEM_α_90P	0.975
CTRW_β_Skewness	0.927	DKI_K_Energy	0.999	FROC_D_Mean	0.973	IVIM_D_Skewness	0.952	SEM_α_Energy	0.997
CTRW_β_TE	0.998	DKI_K_Entropy	0.914	FROC_D_Median	0.980	IVIM_D_TE	0.988	SEM_α_Entropy	0.908
CTRW_β_Uniformity	0.949	DKI_K_IR	0.955	FROC_D_Minimum	0.938	IVIM_D_Uniformity	0.973	SEM_α_IR	0.948
CTRW_β_Variance	0.918	DKI_K_Kurtosis	0.779	FROC_D_Range	0.923	IVIM_D_Variance	0.905	SEM_α_Kurtosis	0.614
CTRW_D_10P	0.957	DKI_K_Maximum	0.909	FROC_D_RMAD	0.900	IVIM_D*_10P	0.969	SEM_α_Maximum	0.943
CTRW_D_90P	0.941	DKI_K_MAD	0.965	FROC_D_RMS	0.960	IVIM_D*_90P	0.974	SEM_α_MAD	0.927
CTRW_D_Energy	0.983	DKI_K_Mean	0.987	FROC_D_Skewness	0.957	IVIM_D*_Energy	0.995	SEM_α_Mean	0.983
CTRW_D_Entropy	0.962	DKI_K_Median	0.901	FROC_D_TE	0.981	IVIM_D*_Entropy	0.955	SEM_α_Median	0.845
CTRW_D_IR	0.920	DKI_K_Minimum	0.923	FROC_D_Uniformity	0.962	IVIM_D*_IR	0.974	SEM_α_Minimum	0.883
CTRW_D_Kurtosis	0.947	DKI_K_Range	0.914	FROC_D_Variance	0.895	IVIM_D*_Kurtosis	0.806	SEM_α_Range	0.905

Table S4 Continued

Feature	ICC	Feature	ICC	Feature	ICC	Feature	ICC	Feature	ICC
SEM_α_RMAD	0.942	SEM_DDC_Entropy	0.955	SEM_DDC_RMAD	0.934	DWI_{conv}_ADC_Entropy	0.962	DWI_{conv}_ADC_RMAD	0.898
SEM_α_RMS	0.984	SEM_DDC_IR	0.932	SEM_DDC_RMS	0.962	DWI_{conv}_ADC_IR	0.901	DWI_{conv}_ADC_RMS	0.961
SEM_α_Skewness	0.895	SEM_DDC_Kurtosis	0.916	SEM_DDC_Skewness	0.952	DWI_{conv}_ADC_Kurtosis	0.951	DWI_{conv}_ADC_Skewness	0.967
SEM_α_TE	0.997	SEM_DDC_Maximum	0.950	SEM_DDC_TE	0.982	DWI_{conv}_ADC_Maximum	0.930	DWI_{conv}_ADC_TE	0.981
SEM_α_Uniformity	0.929	SEM_DDC_MAD	0.943	SEM_DDC_Uniformity	0.961	DWI_{conv}_ADC_MAD	0.907	DWI_{conv}_ADC_Uniformity	0.962
SEM_α_Variance	0.905	SEM_DDC_Mean	0.971	SEM_DDC_Variance	0.942	DWI_{conv}_ADC_Mean	0.972	DWI_{conv}_ADC_Variance	0.901
SEM_DDC_10P	0.985	SEM_DDC_Median	0.977	DWI_{conv}_ADC_10P	0.991	DWI_{conv}_ADC_Median	0.980		
SEM_DDC_90P	0.948	SEM_DDC_Minimum	0.953	DWI_{conv}_ADC_90P	0.933	DWI_{conv}_ADC_Minimum	0.941		
SEM_DDC_Energy	0.982	SEM_DDC_Range	0.954	DWI_{conv}_ADC_Energy	0.981	DWI_{conv}_ADC_Range	0.937		

ICC, intraclass correlation coefficient; CTRW, continuous time random walk; DKI, diffusion kurtosis imaging; FROC, fractional-order calculus; IVIM, intravoxel incoherent motion; MEM, mono-exponential model; ADC, apparent diffusion coefficient; SEM, stretched exponential model; P, percentile; IR, interquartile range; MAD, mean absolute deviation; RMAD, robust mean absolute deviation; RMS, root mean squared; TE, total energy

Appendix E3 Comparison of LASSO selected metrics

Table S5 Comparison of LASSO selected metrics for predicting muscle invasion

Metrics	training cohort			testing cohort		
	NMIBC	MIBC	*p*	NMIBC	MIBC	*p*
CTRW						
α-skewness	-2.34 (-2.96, -1.74)	-1.09 (-1.65, -0.73)	0.000	-1.39 (-1.94, -0.77)	-1.13 (-1.59, -0.95)	0.769
D-mean†	2.12 (1.87, 2.42)	1.45 (1.28, 1.56)	0.000	2.26 (1.90, 2.56)	1.65 (1.39, 1.82)	0.000
D-skewness	0.96 (0.56, 1.29)	1.56 (1.20, 2.02)	0.000	0.69 (0.45, 0.92)	1.70 (1.61, 2.38)	0.000
DKI						
D-mean†	2.31 (2.05, 2.69)	1.77 (1.57, 2.06)	0.000	0.78 (0.61, 0.94)	0.64 (0.53, 0.81)	0.118
D-skewness	0.73 (0.39, 1.17)	1.34 (1.00, 1.83)	0.000	0.71 ± 0.43	1.46 ± 0.60	0.000
D-uniformity	0.12 (0.10, 0.15)	0.18 (0.14, 0.26)	0.000	0.10 (0.09, 0.13)	0.18 (0.14, 0.25)	0.000
K-median†	0.59 ± 0.13	0.74±0.13	0.000	0.50 (0.42, 0.60)	0.65 (0.56, 0.70)	0.000
FROC						
D-90P†	2.34 (2.51, 2.98)	1.84 (1.55, 2.11)	0.000	2.35 (2.07, 2.67)	2.00 (1.56, 2.25)	0.003
D-mean†	1.50 (1.30, 1.76)	1.12 (0.98, 1.27)	0.000	1.50 (1.24, 1.78)	1.23 (1.07, 1.42)	0.003
D-skewness	1.02 (0.62, 1.40)	1.69 (1.35, 2.14)	0.000	0.90 (0.49, 1.24)	2.01 (1.67, 2.34)	0.000
μ-uniformity	0.17 (0.13, 0.22)	0.28 (0.20, 0.33)	0.000	0.14 (0.10, 0.18)	0.20 (1.67, 2.34)	0.018
IVIM						
D-kurtosis	3.74 (3.06, 4.79)	5.86 (4.36,7.83)	0.000	3.32 (2.78, 4.04)	4.06 (3.41, 6.43)	0.004
D-median†	1.03 (0.88, 1.28)	0.76 (0.68, 0.89)	0.000	1.11 (0.96, 1.34)	0.83 (0.75, 0.90)	0.000
D-skewness	0.82 ± 0.13	1.52 ± 0.64	0.000	-0.17 (-0.47, 0.46)	1.02 (-0.03, 1.26)	0.000
D*-uniformity	0.17 (0.12, 0.27)	0.30 (0.19, 0.42)	0.000	0.18 (0.11, 0.34)	0.33 (0.16, 0.43)	0.093
f-skewness	0.27 (-0.01, 0.53)	0.61 (0.33, 0.87)	0.000	0.20 (0.05, 0.48)	0.48 (0.25, 0.79)	0.014
SEM						
α-energy‡	1.69 (0.83, 4.00)	7.74 (3.46, 20.40)	0.000	1.39 (0.61, 3.19)	11.7 (5.07, 26.2)	0.000
α-TE‡	1.70 (0.83, 4.01)	7.74 (3.46, 20.39)	0.000	1.39 (0.62, 3.19)	11.7 (5.07, 26.2)	0.000
DDC-mean†	2.02 (1.78, 2.43)	1.53 (1.33, 1.83)	0.000	1.70 (1.63, 2.24)	1.68 (1.50, 2.04)	0.038
DDC-median†	1.80 (1.54, 2.27)	1.29 (1.09,1.48)	0.000	1.76 (1.44, 2.08)	1.41 (1.18, 1.73)	0.005
DDC-skewness	1.01 (0.57, 1.32)	1.72 (1.31, 2.24)	0.000	0.93 ± 0.61	1.84 ± 0.85	0.000
DWI_{conv}						
ADC-90P†	2.74 (2.35, 3.07)	2.05 (1.80, 2.49)	0.000	2.33 (1.94, 2.75)	2.53 (2.29, 2.78)	0.237
ADC-mean†	1.72 (1.49, 2.02)	1.28 (1.14, 1.46)	0.000	1.68 (1.42, 1.95)	1.41 (1.25, 1.64)	0.003
ADC-skewness	0.99 ± 0.58	1.73 ± 0.62	0.000	0.77 ± 0.49	1.59 ± 0.61	0.000

CTRW, continuous time random walk; DKI, diffusion kurtosis imaging; FROC, fractional-order calculus; IVIM, intravoxel incoherent motion; MEM, mono-exponential model; SEM, stretched exponential model; DWI, diffusion-weighted imaging; ADC, apparent diffusion coefficient; 90P, 90th percentile; TE, total energy

Table S6 Comparison of LASSO selected metrics for predicting histological grade of NMIBC

Metrics	Training cohort			Testing cohort		
	LG-NMIBC	HG-NMIBC	*p*	LG-NMIBC	HG-NMIBC	*p*
CTRW						
β-median†	0.82 (0.76, 0.87)	0.77 (0.70, 0.83)	0.000	0.89 (0.82, 0.99)	0.80 (0.75, 0.88)	0.028
β-skewness	-0.50 (-0.93, -0.16)	-0.31 (-0.65, -0.12)	0.001	-0.90 (-1.66, -0.14)	-0.30 (-0.79, -0.18)	0.210
D-10P†	1.22 (1.02, 1.56)	1.05 (0.82, 1.30)	0.000	1.15 (0.90, 1.40)	1.02 (0.81, 1.21)	0.085
DKI						
K-mean†	0.54 (0.47, 0.61)	0.66 (0.60, 0.75)	0.000	0.44 ± 0.16	0.63 ± 0.13	0.000
K-median†	0.54 (0.47, 0.62)	0.67 (0.60, 0.76)	0.000	0.45 (0.39, 0.54)	0.60 (0.52, 0.63)	0.000
FROC						
β-median†	0.82 (0.77, 0.86)	0.74 (0.71, 0.81)	0.000	0.88 (0.82, 0.98)	0.81 (0.75, 0.87)	0.014
β-skewness	-0.54 (-1.09, -0.10)	-0.07 (-0.53, 0.14)	0.000	-0.94 (-1.59, -0.18)	-0.49 (-0.78, -0.19)	0.304
IVIM						
D-median†	1.15 (0.95, 1.37)	0.90 (0.77, 1.01)	0.000	1.28 ± 0.25	0.96 ± 0.25	0.000
f-10P	0.14 (0.09, 0.20)	0.26 (0.19, 0.31)	0.000	0.02 (0.00, 0.05)	0.12 (0.06, 0.34)	0.000
SEM						
α-median†	0.79 (0.75, 0.83)	0.74 (0.69, 0.78)	0.000	0.84 (0.78, 0.93)	0.78 (0.71, 0.83)	0.013
α-skewness	-0.50 (-1.16, -0.05)	-0.11 (-0.57, 0.11)	0.000	-0.56 (-1.34, -0.13)	-0.33 (-0.65, -0.05)	0.188
DDC-10P	1.17 (0.96, 1.58)	1.02 (0.75, 1.29)	0.000	1.12 ± 0.52	0.85 ± 0.43	0.054
DWI_{conv}						
ADC-10P	1.07 (0.90, 1.41)	0.91 (0.72, 1.08)	0.000	1.12 ± 0.42	0.87 ± 0.29	0.021
ADC-median†	5.81 (1.40, 1.97)	1.38 (1.19, 1.62)	0.000	1.66 (1.42, 1.92)	1.28 (1.19, 0.55)	0.001
ADC-skewness	0.91 ± 0.57	1.14 ± 0.56	0.006	0.66 ± 0.51	1.01 ± 0.33	0.007

CTRW, continuous time random walk; DKI, diffusion kurtosis imaging; FROC, fractional-order calculus; IVIM, intravoxel incoherent motion; MEM, mono-exponential model; SEM, stretched exponential model; DWI, diffusion-weighted imaging; ADC, apparent diffusion coefficient; 10P = 10th percentile
